# Supplementary material for: COVID-19 Vaccination Status as Well as Factors Associated with COVID-19 Vaccine Acceptance and Hesitancy among Prisoners and the Implications
Source: Vaccines (Basel). 2023 Jun 9;11(6):1081. doi: 10.3390/vaccines11061081 (PMC10301173; doi:10.3390/vaccines11061081)
Supplement: Supplementary file 1 [file vaccines-11-01081-s001.zip › vaccines-2378471-supplementary.pdf]

COVID-19 Vaccination Status as Well as Factors Associated with COVID-19 Vaccine Acceptance and Hesitancy among Prisoners and the Implications

Supplementary Material

<u>Section 1 Demographics details</u>

Q1 Gender

1 Male

2 Female

Q2 Age (Years)

1 Below 18

2 18–44

3 45–64

4 Above 65

Q3 Marital status

1 Single/divorced/widow

2 Married

Q4 Sons/daughters

1 Yes

2 No

Q5 Housing type

1 single cell

2 shared cell

Q6 Education level?

1 Illiterate

2 Religious education

3 Primary education

4 High education

Q7 Occupation before detention

1 Employed

2 Unemployed

Q8 Working activity in the prison

1 Yes

2 No

Q9 Having generalized anxiety/depression symptoms

1 Yes

2 No

Q10 Influenza vaccination status

1 Vaccinated

2 Not vaccinated

Q11 Hepatitis B vaccination status

1 Vaccinated

2 Not vaccinated

Q12 Family member get infected with COVID-19

1 Yes

2 No

Q13 Family member died with COVID-19

1 Yes

2 No

Q14 Did you get covid-19 Vaccine?

1 Yes

2 No

Q15 If yes how many doses

1 One

2 One + two

3 One+two+ booster

<u>Section 2: Reasons for vaccines acceptance</u>

What were the reasons to get/accept COVID-19 vaccines? (You can select multiple options).

Sr. No.	Reasons of COVID-19 vaccine acceptance	Yes	No
1	I didn’t fear possible side effects of the COVID-19 vaccines		
2	The vaccination is for free		
3	The desire to return to normal life as soon as possible		
4	I want to participate fight against COVID-19		
5	I have no doubt about the safety of COVID-19 vaccines		
6	Fear of getting COVID-19		
7	Fear of transmitting COVID-19 to others		
8	I am at high risk of acquiring COVID-19 infection		
9	Government makes it compulsory for everyone to get vaccine		

If you didn’t get vaccines yet, are you willing to get it?

1 Yes

2 No

What are the reasons for your willingness to get COVID-19 vaccines.

………

If you are not willing to get COVID-19 what are reasons for it?

<u>Section 3: Reasons for vaccines hesitancy</u>

What were the reasons not to get/accept COVID-19 vaccines? (You can select multiple options).

Sr. No.	Reasons of COVID-19 vaccine hesitancy	Yes	No
1	I am concerned about the side effects of the COVID19 vaccine		
2	I don't feel comfortable with vaccines in general		
3	I am concerned that the vaccine will not work		
4	I don't think I need it		
5	I heard or read news that COVID19 vaccine is dangerous		

6	I have had a bad prior reaction to another vaccine		
7	I already had COVID19		
8	I believe it is better to get natural infection than a vaccine		
9	I don't believe COVID19 is a real problem		
10	The vaccine is a conspiracy		
11	If lots of other people get the vaccine, I won't need it		
12	Others		
